# Metabolomic Insights into the Mechanisms of Ganoderic Acid: Protection against α-Amanitin-Induced Liver Injury

**DOI:** 10.3390/metabo13111164

**Published:** 2023-11-20

**Authors:** Chong Zheng, Shaofang Lv, Jianfang Ye, Lu Zou, Kai Zhu, Haichang Li, Yongxi Dong, Lei Li

**Affiliations:** 1 Guizhou Provincial Center for Disease Control and Prevention, Guiyang 550004, China; zhengchongvip@gmail.com (C.Z.);; 2 School of Pharmacy, Guizhou Medical University, Guiyang 550025, China; 3 Guiyang Provincial Center for Disease Control and Prevention, Guiyang 550002, China

**Keywords:** α-Amanitin, Ganoderic acid A, metabonomics, intervention treatment

## Abstract

α-Amanitin is a representative toxin found in the *Amanita* genus of mushrooms, and the consumption of mushrooms containing α-Amanitin can lead to severe liver damage. In this study, we conduct toxicological experiments to validate the protective effects of Ganoderic acid A against α-amanitin-induced liver damage. By establishing animal models with different durations of Ganoderic acid A treatment and conducting a metabolomic analysis of the serum samples, we further confirmed the differences in serum metabolites between the AMA+GA and AMA groups. The analysis of differential serum metabolites after the Ganoderic acid A intervention suggests that Ganoderic acid A may intervene in α-amanitin-induced liver damage by participating in the regulation of retinol metabolism, tyrosine and tryptophan biosynthesis, fatty acid biosynthesis, sphingosine biosynthesis, spermidine and spermine biosynthesis, and branched-chain amino acid metabolism. This provides initial insights into the protective intervention mechanisms of GA against α-amanitin-induced liver damage and offers new avenues for the development of therapeutic drugs for α-Amanitin poisoning.

## 1. Introduction

Mushrooms enjoy significant popularity among individuals at the dining table; nevertheless, notwithstanding their delectable nature, mushrooms possess the potential to be a “table killer” [1]. In the case of mushroom poisoning, *Amanita* species are significant causes of death, and the most lethal of *Amanita* toxins is α-Amanitin (AMA) [2,3]. While AMA has been shown to cause damage to both the liver and kidneys, the liver stands out as the predominant affected target organ according to AMA toxicokinetic performance [4]. Furthermore, the main target and its primary biological target are thought to be RNA polymerase II in liver cells [5]. However, other studies have reported that an inhibition of RNA polymerase II by AMA is not the only mechanism by which liver injury can occur [6,7]. Understanding the toxic mechanisms of AMA is of paramount importance for the development of potential therapeutic drugs.

**Figure 1:**
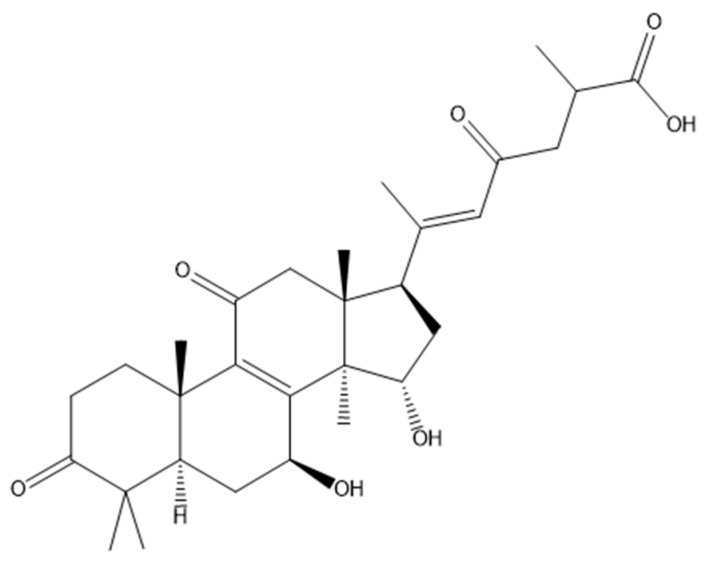
The molecular structure of Ganoderic acid A (GA).

Ganoderic acid A (GA), characterized by the molecular formula C_30_H_44_O_7_ and represented by its molecular structure in Figure 1, is a prominent bioactive compound found in *Ganoderma lucidum*. It boasts a range of pharmacological attributes, including anticancer, antioxidant, and anti-inflammatory properties, which render it extensively employed in various biomedical applications [8]. Furthermore, GA displays promising potential in the therapeutic intervention of liver injuries [9]. Hence, we embarked on utilizing GA to intervene in α-amanitin-induced liver injury. Through a metabolomics analysis, we seek to investigate the intervention mechanism of GA on α-amanitin-induced liver injury, thereby offering novel insights into drug development for the treatment of AMA poisoning.

## 2. Materials and Methods

### 2.1. Chemicals

Ultrapure water was generated using a Milli-Q water-purification system from Milipore (Bedford, MA, USA). Acetonitrile (LC/MS grade) and methanol (LC/MS grade) were purchased from Fisher Scientific (Waltham, MA, USA). Formic acid was bought from Aladdin (Shanghai, China) and α-Amanitin was purchased from Med Chem Express (Shanghai, China). Ganoderic acid A was purchased from Shanghai Yuanye Bio-Technology (Shanghai, China).

### 2.2. Animal Treatment

Male Kunming (KM) mice (18~22 g) were supplied by the Animal Center of Guizhou Medical University (Guizhou, China). The animal usage license number was (SCXK Guizhou) 2018-0001 and the animal quality certificate number was 1202700. Animal welfare and experimental procedures were conducted in accordance with National Institute of Health (NIH) guidelines. The mice were acclimatized for a week at a temperature of 25 ± 2 °C at humidity levels of 50–60% using 12 h light/dark cycles before any animal experiments were performed.

After acclimatization to the environment, the experimental mice were randomly divided into 6 groups, with 6 mice in each group, for the validation experiment of GA treatment on α-amanitin-induced hepatotoxicity. Based on the preliminary research, a dose of 0.3 mg/kg of AMA was used to induce stable pathological indicators in the mouse model [10]. The experimental groups were divided into the control group (a single intraperitoneal injection of physiological saline without AMA), the AMA group (AMA dose of 0.3 mg/kg bw), and the AMA+GA group (after AMA administration, GA was administered at concentrations of 5, 10, 20, and 40 mg/kg bw, respectively). Both AMA and AMA+GA groups received a single intraperitoneal injection of AMA at a concentration of 30 μg/mL, with an injection volume of 0.01 mL/g bw. In the AMA+GA group, after AMA administration, GA was administered via oral gavage at doses of 5, 10, 20, and 40 mg/kg bw immediately and repeated every 6 h. All experimental animals were euthanized 48 h later.

After determining the optimal therapeutic dosage of GA, three groups were established: healthy mice (control), α-amanitin-exposed mice (AMA), and GA-treatment mice (AMA+GA). The dosing regimen for AMA in the control, AMA, and AMA+GA groups was as previously described. Additionally, in the AMA+GA group, GA was administered at a dose of 40 mg/kg of body weight every 6 h, immediately after AMA administration by gavage. Additionally, two separate GA treatment time groups were established at 24 and 48 h. Animals in each group were euthanized after 24 and 48 h of GA treatment, respectively.

### 2.3. Sample Collection and Preparation

The animals were handled using the retro-orbital venous plexus puncture method to extract blood samples. Equal amounts of individual blood samples were combined to create quality-control (QC) samples for each group. All the samples were centrifuged at 2500× *g* for 15 min to separate the serum. After a biochemical analysis of the serum samples, the remaining serum samples were stored at −80 °C, awaiting high-resolution mass spectrometry analysis.

The liver tissues of each mouse were fixed in 10% neutral formalin, dehydrated in a graded manner using a Leica ASP200S automatic tissue dehydrator, and embedded in paraffin. Tissue sections, 5 μm thick, were cut using a Leica RM2255 microtome. The tissue sections were mounted on glass slides and coverslipped using a Leica CV5030 automated coverslipper. The sections were stained with hematoxylin and eosin (H&E) and observed under a Leica DM500 biological microscope to examine tissue pathological changes.

Frozen serum samples for the mass spectrometry analysis were allowed to thaw at room temperature before the analysis. Serum samples of 20 μL were collected and placed in 1.5 mL centrifuge tubes, to which 60 μL of acetonitrile was added to precipitate proteins. After standing for 5 min, the samples were vortexed for 10 min and then centrifuged at 12,000 rpm for 10 min. The supernatant was collected and placed into an inner support tube for UPLC/Q-TOF-MS analysis. The samples were randomly arranged for the collection of mass spectrometry data, with QC sample information collected after every three sample collections.

### 2.4. Oxidative Damage and Inflammatory Markers

A total of 0.5 g of liver tissue was used to prepare a liver homogenate. A total of 1 mL of liver homogenate was mixed with 5 mL of pancreatic enzyme digestion solution and digested in a constant-temperature water bath at 37 °C for 30 min. The digestion was terminated with cold phosphate-buffered saline (PBS). To the prepared tissue suspension, 10 μM of dichlorofluorescein diacetate was added using dimethyl sulfoxide as a solvent control. The mixture was then incubated at 37 °C for 60 min, followed by centrifugation at 1000× *g* for 10 min. The supernatant was discarded and the pellet was washed twice with PBS [11]. The pellet was resuspended in PBS and used for fluorescence detection to measure reactive oxygen species (ROS) indicators. The wavelength settings were as follows: optimal excitation wavelength at 500 nm and optimal emission wavelength at 525 nm. The measurements of interleukin-6 (IL-6), tumor necrosis factor-α (TNF-α), and cyclooxygenase-2 (COX-2) were conducted using the conventional ELISA (enzyme-linked immunosorbent assay) method.

### 2.5. UPLC/Q-TOF-MS Analysis

Chromatographic separations were performed with an Agilent 1290 UHPLC system using an Agilent Zorbax Eclipse Plus C18 column (100 mm × 2.1 mm, 1.8 μm), with elution performed at a flow rate of 0.3 mL/min at 35 °C. In the positive-ion acquisition mode, mobile phase A consisted of 0.1% of formic acid in water, while mobile phase B comprised 0.1% of formic acid in acetonitrile. In the negative-ion acquisition mode, mobile phase A consisted of 5 mmol/L of ammonium acetate in water and mobile phase B contained 5 mmol/L of ammonium acetate in acetonitrile. The gradient elution program was as follows: 0–3.0 min, 5–30% B; 3.0–12.0 min, 30–80% B; 12.0–14.0 min, 80–100% B; and 14.0–18.0 min, 100% B. This elution profile produced an overall elution time of 18 min, which was followed by a post-elution time of 4 min. Aliquots of 2 μL of each supernatant fraction were used for UPLC/Q-TOF-MS for the analysis.

An MS analysis was performed using an Agilent 6545 Q-TOF mass spectrometer (Agilent, Singapore, Singapore) with electrospray ionization in positive and negative modes and a full-scan mode from 50–1000 *m*/*z*. The capillary voltage was set to 4.0 kV in the positive-ion mode and 3.5 kV in the negative-ion mode, using a drying gas flow rate of 12 L/min and a gas temperature of 350 °C. The nebulizer pressure used was 40 psi, with the fragmentor voltage set to 110 V and the skimmer voltage set to 65 V. The MS analysis was performed using a mixture of purine (121.0508 *m*/*z*) and hexakis phosphazine (922.0097 *m*/*z*) as internal standards to ensure mass accuracy for an acquisition time of 500 ms.

### 2.6. Data Processing and Statistical Analysis

Profinder software (version 10.0, Agilent) was used to convert raw data into tables containing pairs of mass and retention time data with associated intensities for all peaks detected. The parameters used in the processing method used a retention time window of 0.2 min and a mass tolerance level of 10 ppm ± 10 mDa. Peaks that were not present in 80% of the samples of a group were filtered out, with peak significance subsequently measured using one-way ANOVA, *t*-tests, fold change analyses, and variable importance in projection (VIP). Metabolites were considered significant when *p* < 0.05 and VIP > 1.0 [12].

### 2.7. Identification of Potential Biomarkers

Metabolites were identified through comparisons with known data present in the Human Metabolome Database (www.hmdb.ca, accessed on 15 August 2022) and METLINE (http://metlin.scripps.edu, accessed on 15 August 2022) for allowable mass window errors of 10 ppm. The metabolic pathways responsible for production were analyzed using MetaboAnalyst 5.0 (https://www.metaboanalyst.ca, accessed on 25 August 2022).

## 3. Results

### 3.1. Blood Biochemical Indicators

In the AMA group, the levels of alanine aminotransferase (ALT), aspartate aminotransferase (AST), alkaline phosphatase (ALP), gamma-glutamyl transpeptidase (GGT), blood urea nitrogen (BUN), and creatinine (Cr) were significantly higher than those in the control group, with a statistical significance of (*p* < 0.05). Compared to the AMA group, animals treated with varying doses of GA exhibited a substantial reduction in serum levels of ALT, AST, ALP, GGT, BUN, and Cr. Furthermore, this reduction demonstrated a noteworthy dose–response relationship (*p* < 0.05) (Table 1). The most optimal therapeutic outcome was observed for a GA treatment dose of 40 mg/kg bw. At the optimal dose of GA (40 mg/kg bw), the animals in each time group exhibited a notable reduction in serum levels of ALT, AST, ALP, GGT, BUN, and Cr when compared to the AMA group. This reduction became more prominent with the prolongation of the treatment duration (*p* < 0.05) (Table 2).

### 3.2. Histopathological Findings for Liver Tissue

**Figure 2:**
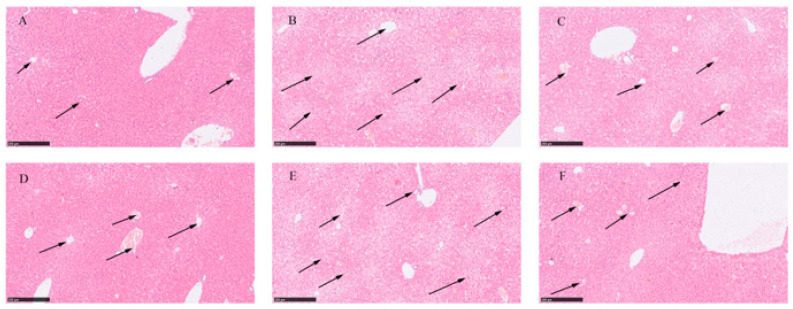
Histopathological images of healthy liver tissue, liver tissue exposed to AMA, and liver tissue after GA treatment. (**A**) Histopathological image of liver tissue at 24 h. (**B**) Histopathological image of liver tissue after 24 h exposure to AMA. (**C**) Histopathological image of liver tissue after 24 h GA treatment. (**D**) Histopathological image of liver tissue at 48 h. (**E**) Histopathological image of liver tissue after 48 h exposure to AMA. (**F**) Histopathological image of liver tissue after 48 h GA treatment.

The liver tissue structure in the 24 and 48 h control groups appeared normal. The hepatic cords and hepatic sinusoids were clearly visible, and the hepatic sinusoids were filled with numerous red blood cells (as indicated by arrows in Figure 2A,D). In the AMA group, the histopathological images of liver tissue specimens showed a disruption of the liver lobule structure, the disappearance of the hepatic cord structure, focal necrosis and swelling of hepatocytes, a significant reduction in the hepatic sinusoidal area, and poor filling of the hepatic sinusoids. There was an evident infiltration of inflammatory cells (mainly mononuclear cells and macrophages) in the liver lobules (as indicated by arrows in Figure 2B,E). Under treatment with GA (40 mg/kg body weight), the severity of liver damage gradually decreased. The liver cord and hepatic sinusoid structures gradually recovered. With the extension of the treatment time, improvements were observed in features, such as hepatic vacuolation, bleeding, infiltration of inflammatory cells (mainly mononuclear cells and macrophages), and hepatocyte necrosis (as indicated by arrows in Figure 2C,F). Under the influence of GA, the progression of liver lesions gradually slowed down, indicating that GA had a certain therapeutic effect on liver damage induced by AMA.

### 3.3. ROS, IL-6, TNF-α, and COX-2 Levels

Compared to the control group, the expression levels of ROS, IL-6, TNF-α, and COX-2 in the liver tissues of mice in the AMA group were significantly increased, and the differences were statistically significant (*p* < 0.05). When comparing the AMA+GA group with the AMA group, the expression levels of ROS, IL-6, TNF-α, and COX-2 were significantly reduced, and this reduction increased with the prolongation of the treatment time (*p* < 0.05) (Table 3).

### 3.4. Multivariate Analysis of Analytes Present in the Blood

Metabolic profiling of the blood samples was conducted using Mass Hunter Workstation Software (version 11.0, Agilent, Bejing, China). After performing the data collection, data preprocessing was performed using Profinder software (Version 10.0, Agilent), which included data retention time alignment, filtering, matching, and peak identification. In the positive-ion mode, 3074 compounds were obtained, and in the negative-ion mode, 2887 compounds were obtained. The relative standard deviation (RSD) of peak areas was calculated using QC samples. Compounds with an RSD higher than 30% were filtered out. The results show that 82% of the peaks in the QC sample group meet the required standards, indicating good stability during the sample analysis process.

**Figure 3:**
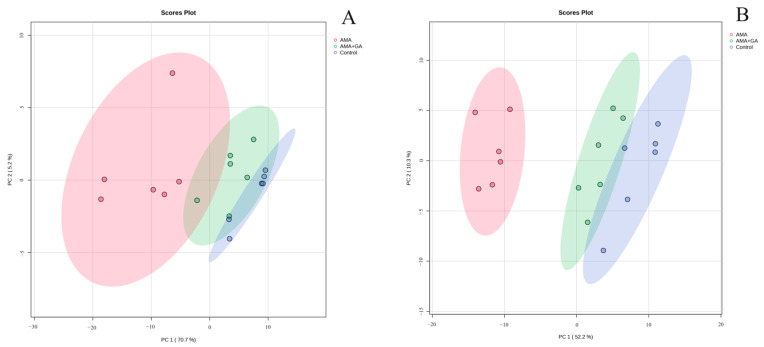
PCA (unsupervised) analysis of serum metabolites in healthy mice (control), α-amanitin-exposed mice (AMA), and GA treatment (AMA+GA). (**A**) Score plot after 24 h of GA treatment. (**B**) Score plot after 48 h of GA treatment.

To comprehensively understand the metabolic differences in the serum samples, the obtained compound data were converted into CEF files and imported into Mass Profiler Professional software (Version 15.1, Agilent) for the multivariate analysis. Statistical filtering was applied to both the 24 and 48 h experimental groups. Compounds with data missing in each group exceeding 20% were filtered out, while compounds exhibiting statistically significant differences between the control group versus the AMA group and the AMA+GA group versus the AMA group (*p* < 0.05) were retained. Following filtering, 116 compounds were obtained for the 24 h experimental group (Supplementary Table S1), and 157 compounds were obtained for the 48 h experimental group (Supplementary Table S2). A Principal component analysis (PCA) model was applied for the clustering analysis of the filtered data. Missing peak response intensities were replaced with the within-group mean. The data were centered around this within-group mean and then normalized by dividing them by the standard deviation of variables within the group. At 24 h, there was a significant separation between the control and AMA groups, while the AMA+GA group was positioned between the control and AMA groups with unclear clustering differentiation and two principal components, including PC 1 (70.7%) and PC 2 (5.2%) in Figure 3A. At 48 h, there was a significant separation between the control and AMA groups, and the AMA+GA group also showed significant differentiation from the AMA group, approaching the control group, resulting in clear clustering distinctions and two principal components, including PC 1 (52.2%) and PC 2 (10.3%) in Figure 3B. Subsequently, the filtered compounds were subjected to clustering analysis once again using the partial least squares discriminant analysis (PLS-DA) model for cluster scoring validation. The results of the PLS-DA analysis further confirm the improved cluster separation for both the 24 and 48 h experimental groups after the GA intervention (Supplementary Figure S1). The results show distinct grouping trends with the increasing intervention time. These results indicate that the process of AMA poisoning disrupts the metabolism of endogenous metabolites, while GA can alter the metabolic disturbances caused by AMA poisoning, thereby exhibiting therapeutic effects. In the 24 and 48 h experimental groups, the filtered compounds’ response intensities were evaluated using the orthogonal partial least squares discriminant analysis (OPLS-DA), and statistical differences in the metabolite response intensities were obtained between the control and AMA groups, as well as between the AMA and AMA+GA groups (Supplementary Figure S2). These findings underscore significant serum metabolic distinctions between the AMA and control groups, as well as noteworthy shifts in serum metabolites following GA treatment compared to the AMA group.

### 3.5. Metabolites’ Identification and Pathway Analysis

Peak response intensities between the control and AMA groups, as well as between the AMA+GA and AMA groups, in the 24 and 48 h experiments were statistically compared. Compounds with significantly altered inter-group peak response intensities, as determined by *t*-tests (*p* < 0.05) and VIP scores higher than 1.0, were considered potential differential metabolites. The selected ions were identified by a comparison with ion data stored in the online Human Metabolome (www.hmdb.ca, accessed on 25 August 2022) and METLINE (https://metlin.scripps.edu, accessed on 25 August 2022) databases. The mass tolerances between the measured *m*/*z* values and exact masses of the compounds were set to 10 ppm. The mass spectrometry information for potential differential metabolites can be found in Table 4. The statistical results of the peak response intensities for the differential metabolites between the control and AMA groups can be found in Supplementary Table S3. The statistical results of the peak response intensities for the differential metabolites between the AMA and AMA+GA groups after the GA intervention are presented in Supplementary Table S4.

**Figure 4:**
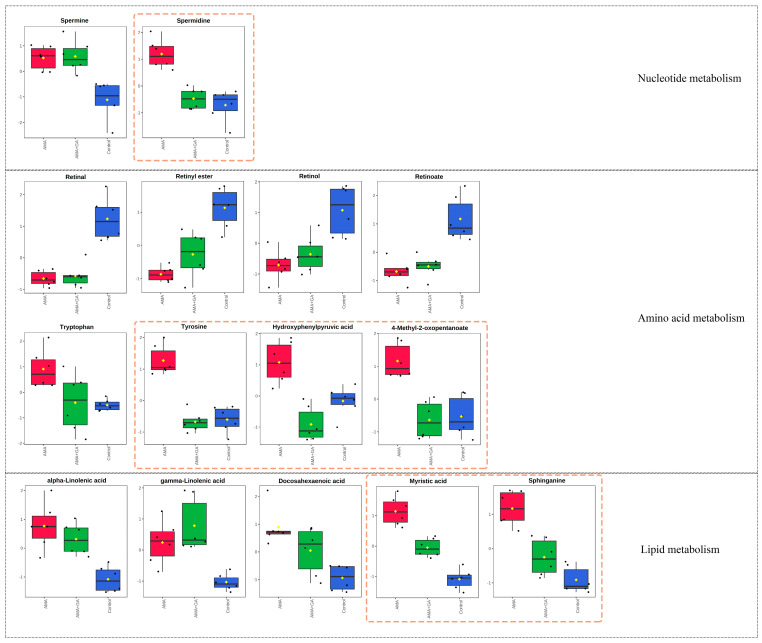
Fifteen metabolites with significant differences (*p* < 0.05, VIP > 1) in the 24 h experimental group after identification, involving metabolic pathways related to nucleotide metabolism, amino acid metabolism, and lipid metabolism. Among these metabolites, spermidine, tyrosine, hydroxyphenylpyruvic acid, 4-methyl-2-oxopentanoate, myristic acid, and sphinganine exhibited significant changes after GA treatment, as highlighted by the yellow dashed boxes in the figure. Red boxes represent the AMA group, green boxes represent the AMA+GA group, blue boxes represent the control group, and yellow dots represent the mean peak response intensity of compounds within each group.

The analysis of differential metabolites in the 24 h experimental group revealed 15 identified differential metabolites, mainly including amino acids, fatty acids, sphingolipids, and other compounds. Among these, six compounds exhibited significant changes during the early stages of GA treatment (*p* < 0.05, VIP > 1). This suggests that GA can modulate the metabolic abnormalities induced by AMA during the initial treatment, particularly affecting metabolites, like spermidine, sphinganine, myristic acid, tyrosine, 4-methyl-2-oxopentanoate, and hydroxyphenylpyruvic acid (Figure 4). Subsequently, we conducted a metabolic pathway enrichment analysis using MetaboAnalyst 5.0. The enrichment analysis showed that, in the early stages of GA treatment, there was a significant regulation (*p* < 0.05) of spermidine and spermine biosynthesis, tyrosine and tryptophan biosynthesis, sphingolipid metabolism, fatty acid biosynthesis, and branched-chain amino acid metabolism induced by AMA (Supplementary Figure S3).

**Figure 5:**
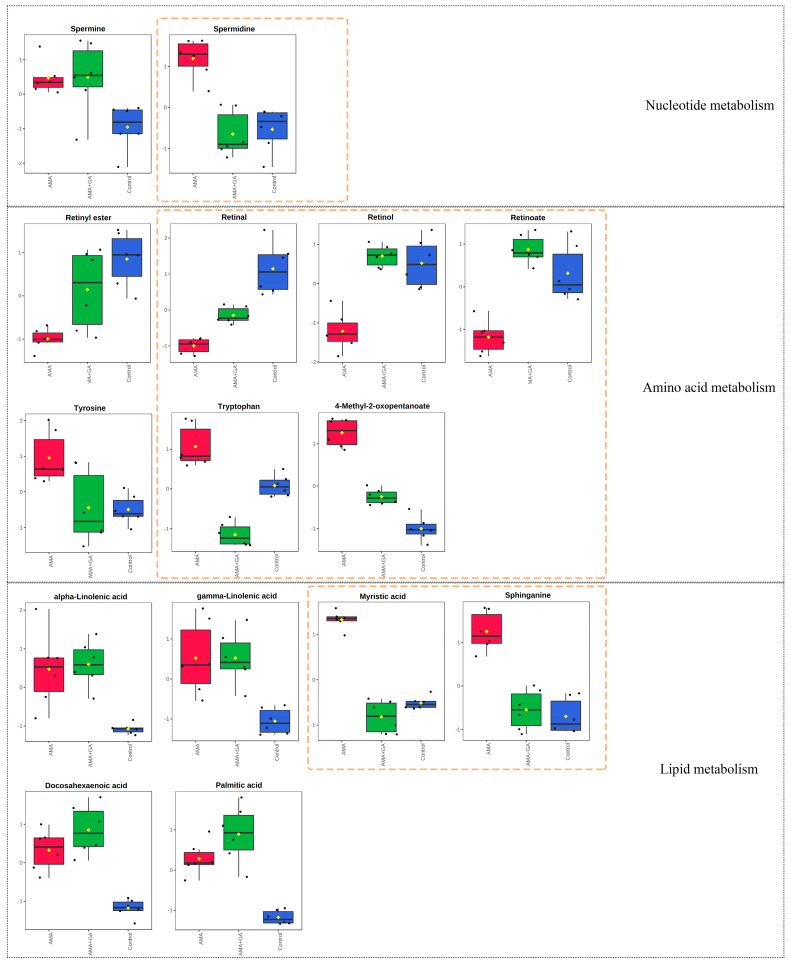
Fifteen metabolites with significant differences (*p* < 0.05, VIP > 1) in the 48 h experimental group after identification, encompassing metabolic pathways associated with nucleotide metabolism, amino acid metabolism, and lipid metabolism. Notably, the compounds spermidine, retinal, retinol, retinoate, tryptophan, 4-methyl-2-oxopentanoate, myristic acid, and sphinganine exhibited substantial changes following GA treatment, as highlighted by the yellow dashed boxes in the figure. Red boxes represent the AMA group, green boxes represent the AMA+GA group, blue boxes represent the control group, and yellow dots represent the mean peak response intensity of compounds within each group.

Subsequently, an analysis of differential metabolites in the 48 h experimental group identified 15 differential metabolites. Among these, eight compounds exhibited significant changes during the mid-term GA treatment (*p* < 0.05, VIP > 1). This indicated that, in the mid-term of the treatment, GA could modulate the metabolic abnormalities caused by AMA, including retinal, retinol, retinoate, spermidine, sphinganine, tryptophan, myristic acid, and 4-methyl-2-oxopentanoate (Figure 5). The metabolic pathway enrichment analysis was conducted using MetaboAnalyst 5.0. The enrichment analysis demonstrated that after GA treatment, several metabolic pathways induced by AMA, including retinol metabolism, tyrosine and tryptophan biosynthesis, fatty acid biosynthesis, sphingosine biosynthesis, spermidine and spermine biosynthesis, and branched-chain amino acid metabolism, were significantly modulated (*p* < 0.05) (Supplementary Figure S3).

## 4. Discussion

The hepatotoxicity of AMA primarily arises from its inhibition of RNA polymerase II activity, disrupting normal RNA synthesis in liver cells, ultimately leading to hepatocyte death [13]. However, the inhibition of RNA polymerase II is not the sole pathway through which α-amanitin-induced liver damage occurs [14,15,16]. There may be other therapeutic targets for α-amanitin-induced liver injury. *Ganoderma lucidum* (Lingzhi) has a long history of medicinal use in China, and GA is one of the major triterpenoid compounds found in Lingzhi [17]. It has shown a protective effect against liver damage [18]. In this study, we aimed to validate the protective role of GA against α-amanitin-induced liver injury using an animal model, and to explore the metabolic pathways regulated by GA during the treatment process through a metabolomics analysis.

Serum biochemical markers can reflect the body’s metabolic function and tissue damage status [19]. Among them, alterations in AST, ALT, and ALP levels are indicative of liver tissue damage [20,21], while GGT concentrations are utilized for liver injury diagnosis [22]. In our study, mice exposed to AMA exhibited a notable elevation (*p* < 0.05) in serum levels of ALT, AST, ALP, and GGT. Furthermore, a histopathological examination of liver specimens confirmed disruptions in the hepatic lobular structure, focal necrosis, hepatocyte swelling, and inflammatory cell infiltration induced by AMA. Following GA treatment, there was a substantial reduction (*p* < 0.05) in serum levels of ALT, AST, ALP, and GGT, with these changes becoming more pronounced with an extended GA-treatment duration. Histopathological observations of liver specimens in the AMA+GA group revealed a marked decrease in inflammatory cell infiltration compared to the AMA group, and this amelioration became increasingly significant with prolonged treatment. These findings underscore that GA exhibits therapeutic potential in mitigating liver damage induced by AMA. BUN and Cr levels serve as crucial biochemical markers for diagnosing kidney injury and dysfunction [23,24]. In our study, AMA exposure led to a significant increase (*p* < 0.05) in BUN and Cr levels, while these markers exhibited a substantial decrease (*p* < 0.05) in serum following GA treatment. Furthermore, this decreasing trend became more significant with prolonged treatment, suggesting that GA not only ameliorated liver damage induced by AMA, but also intervened in the renal toxicity induced by AMA.

ROS, IL-6, TNF-α, and COX-2 are biomolecules associated with inflammation and cellular damage. When liver cells are damaged, they can trigger the release of IL-6 and TNF-α by immune cells, promoting an inflammatory response that leads to cell apoptosis [25,26]. Increased COX-2 expression accelerates prostaglandin synthesis, further exacerbating the inflammatory process in liver cells [27]. In addition, the expression of inflammatory factors resulting from liver cell damage can promote the production of more ROS, leading to oxidative stress damage and further exacerbating cell apoptosis [28]. In this study, α-amanitin-treated mice showed a significant increase in ROS, IL-6, TNF-α, and COX-2 levels in serum (*p* < 0.05). Combined with histopathological images of liver specimens, this indicated that mice exhibited an inflammatory response in the liver under the influence of AMA. Following treatment with GA, there was a significant decrease in ROS, IL-6, TNF-α, and COX-2 levels (*p* < 0.05). This, in conjunction with the histopathological results of the liver specimens, suggests that GA can significantly reduce the inflammatory response in liver cells induced by AMA and mitigate the oxidative stress in liver cells, thereby exerting a protective effect on the liver.

PCA models can reflect differences in metabolites between different experimental groups and present the trends in metabolite changes under different intervention times. In this study, PCA models were used to analyze the serum metabolic trends at different intervention times during the process of liver damage induced by AMA and treated with GA. Comparing the serum metabolite PCA clustering results for 24 and 48 h under GA intervention, at 24 h, there was differentiation between the control and AMA groups, but the AMA+GA group was positioned between the two groups and not distinctly separated from the AMA group. However, at 48 h, the AMA+GA group could be significantly distinguished from the AMA group. After GA treatment, the metabolic characteristics of the AMA+GA group gradually approached those of the control group, demonstrating that a GA intervention could regulate the metabolic abnormalities caused by AMA.

The results of the analysis of the metabolic differences between the control and AMA groups indicate that AMA induces relatively minor differences in metabolites at different time points. These differences all point to similar metabolic pathways, primarily involving abnormalities in retinol metabolism, spermidine and spermine metabolism, sphingolipid metabolism, fatty acid metabolism, and unsaturated fatty acid metabolism. Following the GA intervention, it was observed that GA participated in the regulation of retinol metabolism, tyrosine and tryptophan biosynthesis, fatty acid biosynthesis, sphingosine biosynthesis, spermidine and spermine biosynthesis, and branched-chain amino acid metabolism. Retinal, retinol, and retinoate are different forms of vitamin A metabolism. The experimental results indicate that, under the influence of AMA, the concentrations of retinal, retinyl ester, retinol, and retinoate in the serum samples of the experimental animals decrease (*p* < 0.05), which may lead to increased oxidative stress [29], subsequently accelerating the accumulation of ROS within the cells and triggering cell apoptosis [30,31]. Moreover, vitamin A can modulate intracellular lipid metabolism. The decreased levels of different forms of vitamin A compounds can disrupt intracellular lipid body metabolism, promoting lipid body accumulation [32]. Intracellular lipid body accumulation can induce the further aggravation of liver inflammation, hastening liver cell apoptosis [33]. Under GA intervention conditions, significant alterations (*p* < 0.05) were observed in the concentrations of retinal, retinol, and retinoate. This indicates that GA can ameliorate the cellular oxidative environment by regulating different forms of vitamin A compounds, reducing oxidative stress. Simultaneously, GA can regulate lipid metabolism through retinal, retinol, and retinoate, lowering the risk of liver cell inflammation.

Spermidine and spermine are polyamine compounds that are involved in the interaction between DNA and proteins, regulating cell proliferation and differentiation [34]. Maintaining normal levels of polyamines in the body is crucial. Cell damage can lead to an increase in polyamine levels in the body, and excessively high levels of polyamines can produce hydrogen peroxide and acrolein during polyamine metabolism, increasing oxidative stress damage. This can disrupt proteins, DNA, and other cellular components, causing severe toxic damage [35,36]. The experimental results indicate that, under the influence of AMA, the concentrations of spermidine and spermine in the serum samples of the experimental animals significantly increase (*p* < 0.05). This leads to an exacerbation of oxidative stress, and the excess metabolites of polyamines may cause damage to liver cells, accelerating the process of liver cell apoptosis. Under GA intervention conditions, the concentrations of spermidine and spermine both decreased, with spermidine showing the most significant decrease. This suggests that GA can intervene in the polyamine metabolism process in the organism, promoting the return of abnormal polyamine concentration levels caused by AMA to normal values. It reduces oxidative stress levels and the cytotoxic effects of excess polyamine metabolites, thus playing a protective role in the liver.

Sphinganine belongs to sphingolipid compounds, and sphingolipids play a crucial role in membrane and lipoprotein structures, as well as in cellular regulation, serving as growth factors, differentiation factors, cytokines, and, increasingly, as second messengers for various stimuli. Therefore, maintaining stable concentrations of sphingolipids in the body is of great significance [37]. Disruptions in the metabolism of sphinganine-type sphingolipids can lead to cellular toxicity [38,39]. The experimental results indicate that, under the influence of AMA, the concentration levels of sphinganine in the serum samples of the experimental animals significantly increase (*p* < 0.05), leading to cellular toxicity and accelerating apoptosis in liver cells. Under GA intervention conditions, the concentration levels of sphinganine decreased and approached those of the control group. This suggests that GA can effectively regulate the disrupted metabolism of sphingolipids caused by AMA, promoting the return of sphinganine to normal concentration levels and reducing the cytotoxic effects caused by its disruption.

Myristic and palmitic acids are saturated fatty acids that play important roles in the normal metabolism of liver cells [40]. Changes in the concentration levels of saturated fatty acids in the body can affect the oxidative stress levels in liver cells. Specifically, the excessive accumulation of high-concentration saturated fatty acids in liver cells can directly lead to mitochondrial dysfunction and oxidative stress responses, accelerating apoptosis in liver cells [41]. The experimental results indicate that, under the influence of AMA, the serum samples of the experimental animals show a significant increase (*p* < 0.05) in the concentration of saturated fatty acids, represented by myristic and palmitic acids. This leads to an abnormal mitochondrial function in liver cells. Under GA intervention conditions, there was a significant decrease (*p* < 0.05) in the concentration of myristic acid. This suggests that GA can regulate the metabolism of saturated fatty acids and, in turn, the metabolic dysfunction of mitochondria in liver cells. This regulation leads to a reduction in oxidative stress levels in liver cells, ultimately protecting them.

Tyrosine and tryptophan are essential amino acids in the body that play crucial roles in protein synthesis. They are also precursors to various bioactive molecules. Hydroxyphenylpyruvic acid is one of the metabolites of tyrosine. Tyrosine is primarily metabolized in the liver, and severe liver damage can lead to an increase in the concentration of tyrosine and its metabolites [42]. Additionally, abnormally elevated levels of tyrosine in the body can lead to the accumulation of toxic metabolic intermediates, exacerbating liver damage [43]. The experimental results indicate that, under the influence of AMA, there is a significant increase (*p* < 0.05) in the concentrations of tryptophan, tyrosine, and hydroxyphenylpyruvic acids in the serum samples of the experimental animals. This suggests that liver damage caused by AMA disrupts the metabolism of tyrosine and tryptophan pathways. Under GA intervention conditions, there was a significant decrease (*p* < 0.05) in the concentrations of tyrosine and hydroxyphenylpyruvic acids in the 24 h AMA+GA group, and a significant decrease (*p* < 0.05) in tryptophan concentration in the 48 h intervention group. This indicates that a GA intervention can regulate the amino acid metabolism abnormalities caused by AMA.

Methyl-2-oxopentanoate is an intermediate product in the metabolism pathway of valine, and it is involved in branched-chain amino acid metabolism. Branched-chain amino acids are a critical group of amino acids that play essential roles in protein synthesis and energy metabolism [44]. Under the influence of AMA, there is an increase (*p* < 0.05) in the concentration of 4-methyl-2-oxopentanoate, indicating that AMA may interfere with the metabolism of branched-chain amino acids. Branched-chain amino acids are involved in important energy metabolism pathways in the body, and abnormalities in their metabolism can lead to mitochondrial dysfunction in liver cells, resulting in disrupted energy metabolism [45]. Under GA intervention conditions, there was a significant decrease (*p* < 0.05) in the concentration of 4-methyl-2-oxopentanoate, and it returned to normal levels. This suggests that GA can regulate the abnormal metabolism of branched-chain amino acids induced by AMA.

## 5. Conclusions

In summary, we utilized the UPLC-Q/TOF-MS analysis in combination with a pathological analysis to confirm that GA can intervene in liver damage caused by AMA. Observing the trends and differences in metabolites at different stages of the GA intervention suggests that GA can intervene in the liver damage caused by AMA by regulating retinol metabolism, tyrosine and tryptophan biosynthesis, fatty acid biosynthesis, sphingosine biosynthesis, spermidine and spermine biosynthesis, and branched-chain amino acid metabolism. Our findings elucidate the protective intervention mechanisms of GA against α-amanitin-induced liver damage, presenting the possibility of GA as a potential therapeutic target for AMA poisoning.

## Figures and Tables

**Table 1:** Comparison of blood biochemical indicators for the treatment of α-amanitin-induced liver injury with different doses of GA (mean ± SD, *n* = 6, 48 h).

Group	ALT (U/L)	AST (U/L)	ALP (U/L)	GGT (U/L)	BUN (mmol/L)	Cr (μmol/L)
Control	38.50 ± 4.83	61.26 ± 4.15	61.23 ± 6.25	4.36 ± 1.27	8.23 ± 0.82	32.45 ± 2.83
AMA	262.98 ± 34.28 ^a^	315.12 ± 42.13 ^a^	333.25 ± 56.47 ^a^	59.52 ± 10.92 ^a^	100.02 ± 11.28 ^a^	130.82 ± 18.43 ^a^
AMA+GA (5 mg/kg bw)	141.39 ± 15.45 ^b^	258.62 ± 20.98 ^b^	265.50 ± 34.58 ^b^	40.46 ± 9.83 ^b^	85.92 ± 10.52 ^b^	109.35 ± 14.33 ^b^
AMA+GA (10 mg/kg bw)	99.73 ± 8.41 ^bc^	200.62 ± 43.91 ^bc^	200.37 ± 23.21 ^bc^	33.39 ± 2.90 ^bc^	60.59 ± 9.66 ^bc^	87.75 ± 11.26 ^bc^
AMA+GA (20 mg/kg bw)	79.55 ± 9.74 ^bcd^	138.88 ± 14.30 ^bcd^	178.62 ± 36.17 ^bcd^	24.20 ± 2.03 ^bcd^	40.56 ± 4.65 ^bcd^	67.90 ± 11.28 ^bcd^
AMA+GA (40 mg/kg bw)	46.61 ± 9.95 ^bcde^	106.62 ± 10.42 ^bcde^	142.38 ± 22.13 ^bcde^	13.71 ± 2.18 ^bcde^	15.76 ± 5.53 ^bcde^	51.01 ± 1.62 ^bcde^

Note. ^a^ *p* < 0.05 compared to control. ^b^ *p* < 0.05 compared to AMA. ^c^ *p* < 0.05 compared to AMA+GA (5 mg/kg bw). ^d^ *p* < 0.05 compared to AMA+GA (10 mg/kg bw). ^e^ *p* < 0.05 compared to AMA+GA (20 mg/kg bw).

**Table 2:** Comparison of blood biochemical indicators for the treatment of α-amanitin-induced liver injury with different administration times (mean ± SD, *n* = 6).

Group	ALT (U/L)	AST (U/L)	ALP (U/L)	GGT (U/L)	BUN (mmol/L)	Cr (μmol/L)
Control	40.92 ± 6.94	72.75 ± 6.34	55.62 ± 11.72	4.84 ± 0.46	5.64 ± 0.70	35.41 ± 1.98
AMA (24 h)	157.24 ± 22.72 ^a^	140.86 ± 23.81 ^a^	114.00 ± 11.03 ^a^	19.07 ± 9.76 ^a^	12.93 ± 2.71 ^a^	80.67 ± 10.92 ^a^
AMA+GA (24 h)	51.21 ± 16.14 ^b^	118.62 ± 11.21 ^b^	74.00 ± 7.82 ^b^	10.39 ± 2.91 ^b^	9.85 ± 0.96 ^b^	49.54 ± 13.07 ^b^
AMA (48 h)	216.65 ± 28.01 ^ab^	192.75 ± 44.87 ^ab^	253.50 ± 15.70 ^ab^	27.24 ± 4.70 ^ab^	23.04 ± 4.47 ^ab^	160.35 ± 26.43 ^ab^
AMA+GA (48 h)	68.46 ± 8.85 ^c^	117.86 ± 15.26 ^c^	101.43 ± 37.66 ^c^	14.74 ± 1.25 ^c^	8.96 ± 0.85 ^c^	54.14 ± 4.91 ^c^

Note. ^a^ *p* < 0.05 compared to control. ^b^ *p* < 0.05 compared to AMA (24 h). ^c^ *p* < 0.05 compared to AMA (48 h).

**Table 3:** The expression levels of ROS, IL-6, TNF-α, and COX-2 after treatment with GA at different times (mean ± SD, *n* = 6).

Group	ROS (pmoL/mL)	IL-6 (μg/mL)	TNF-α (μg/mL)	COX-2 (mg/mL)
Control	40.67 ± 6.14	32.21 ± 7.04	444.67 ± 96.90	113.33 ± 14.71
AMA (24 h)	100.38 ± 9.39 ^a^	103.67 ± 5.20 ^a^	1813.50 ± 84.60 ^a^	218.17 ± 15.97 ^a^
AMA+GA (24 h)	69.52 ± 5.33 ^b^	69.78 ± 7.23 ^b^	793.03 ± 134.86 ^b^	152.67 ± 16.79 ^b^
AMA (48 h)	202.65 ± 13.85 ^ab^	171.67 ± 11.91 ^ab^	3749.48 ± 109.50 ^ab^	311.17 ± 15.50 ^ab^
AMA+GA (48 h)	103.50 ± 9.63 ^c^	96.91 ± 14.04 ^c^	1272.83 ± 89.96 ^c^	212.50 ± 11.31 ^c^

Note. ^a^ *p* < 0.05 compared to control. ^b^ *p* < 0.05 compared to AMA (24 h). ^c^ *p* < 0.05 compared to AMA (48 h).

**Table 4:** The mass spectrometry information for potential differential metabolites.

Metabolite	Retention Time (min)	Mass Found	Adducts	Exact Mass	MS/MS Fragments	MS/MS
						CE (eV)
Retinal	11.92	285.2217	[M+H]^+^	284.214	161.0955	20
					175.1479	
					119.0849	
Retinyl ester	13.87	303.2311	[M+H]^+^	302.2246	285.221	20
					135.1157	
					103.0533	
Retinol	14.56	287.2373	[M+H]^+^	286.2297	121.0351	20
					269.2248	
					93.0686	
Retinoate	11.06	301.2167	[M+H]^+^	300.2089	123.1166	20
					161.0963	
					81.0696	
Spermidine	0.68	146.1645	[M+H]^+^	145.1579	72.0809	20
					84.0809	
					112.1122	
Spermine	0.7	203.2234	[M+H]^+^	202.2157	112.1122	20
					129.1389	
					84.0804	
Sphinganine	8.76	302.3058	[M+H]^+^	301.2981	60.0451	20
					284.2949	
					252.2844	
Tryptophan	2.59	205.0967	[M+H]^+^	204.0899	146.0602	20
					118.0649	
					188.0714	
Palmitic acid	14.78	255.2324	[M−H]^−^	256.2402	255.2324	20
Docosahexaenoic acid	13.42	327.2325	[M−H]^−^	328.2402	283.2424	20
					327.2323	
					229.1955	
Myristic acid	12.51	227.2019	[M−H]^−^	228.2089	277.2019	20
					68.9957	
α-Linolenic acid	12.66	277.2174	[M−H^]−^	278.2246	277.2174	20
					59.0138	
γ-Linolenic acid	12.84	277.2177	[M−H]^−^	278.2246	277.2177	20
					59.0141	
Tyrosine	0.92	180.0667	[M−H]^−^	181.0739	119.0547	20
					163.0535	
					72.0099	
4-Methyl-2-oxopentanoate	0.93	129.0562	[M−H]^−^	130.063	129.0562	10
					101.0604	
Hydroxyphenylpyruvic acid	0.92	179.0353	[M−H]^−^	180.0423	179.0353	20
					135.0452	
					106.9578	

## Data Availability

The data presented in this study are available from the corresponding authors upon request. The data are not publicly available due to institutional requests.

## Supplementary Materials

The following supporting information can be downloaded at: https://www.mdpi.com/article/10.3390/metabo13111164/s1, Figure S1. PLS-DA (supervised) analysis of serum metabolites in healthy mice (control), α-amanitin-exposed mice (AMA), and GA treatment (AMA+GA). (A) Score plot after 24 h of GA treatment (R^2^ = 0.998, Q^2^ = 0.935). (B) Score plot after 48 h of GA treatment (R^2^ = 0.999, Q^2^ = 0.903). Figure S2. Orthogonal partial least squares discriminant analysis (OPLS-DA) score plots of serum metabolites in healthy mice (control), α-amanitin-exposed mice (AMA), and GA treatment (AMA+GA). (A) Score of the 24 h control versus AMA groups (R^2^X = 0.639, R^2^Y = 0.849 and Q^2^ = 0.815). (B) Score of the 24 h AMA versus AMA+GA groups (R^2^X = 0.443, R^2^Y = 0.676 and Q^2^ = 0.623). (C) Score of the 48 h control versus AMA groups (R^2^X = 0.563, R^2^Y = 0.952 and Q^2^ = 0.930). (D) Score of the 48 h AMA versus AMA+GA groups (R^2^X = 0.493, R^2^Y = 0.933 and Q^2^ = 0.906). Figure S3. Metabolic pathway analysis results. (A) The 24 h control versus AMA groups. (B) The 24 h AMA versus AMA+GA groups. (C) The 48 h control versus AMA groups. (D) The 48 h AMA versus AMA+GA groups. Table S1. Accurate molecular weight, retention time, and peak response intensity of compounds in the 24 h experimental group after statistical filtration. Table S2. Accurate molecular weight, retention time, and peak response intensity of compounds in the 48 h experimental group after statistical filtration. Table S3. The statistical analysis of differential metabolites between control and AMA groups. Table S4. The statistical analysis of differential metabolites between AMA and AMA+GA groups.

## Abbreviations

The following abbreviations are used in this manuscript:

ALP	Alkaline Phosphatase
ALT	Alanine Aminotransferase
AMA	α-Amanitin
AST	Aspartate Aminotransferase
BUN	Blood Urea Nitrogen
COX-2	Cyclooxygenase-2
Cr	Creatinine
GA	Ganoderic Acid A
GGT	Gamma-Glutamyl Transpeptidase
IL-6	Interleukin-6
OPLS-DA	Orthogonal Partial Least Squares Discriminant Analysis
PBS	Phosphate Buffered Saline
PCA	Principal Component Analysis
PLS-DA	Partial Least Squares Discriminant Analysis
QC	Quality Control Samples
ROS	Reactive Oxygen Species
RSD	Relative Standard Deviation
TNF-α	Tumor Necrosis Factor-α
VIP	Variable Importance in Projection
