# Supplementary material for: Metabolomic Insights into the Mechanisms of Ganoderic Acid: Protection against α-Amanitin-Induced Liver Injury
Source: Metabolites. 2023 Nov 20;13(11):1164. doi: 10.3390/metabo13111164 (PMC10672867; doi:10.3390/metabo13111164)
Supplement: Supplementary file 1 [file metabolites-13-01164-s001.zip › Supplementary Figure S1 to S3.pdf]

Supplementary Materials

Metabolomic Insights into the Mechanisms of Ganoderic Acid A Protection Against α-Amanitin-Induced Liver Injury

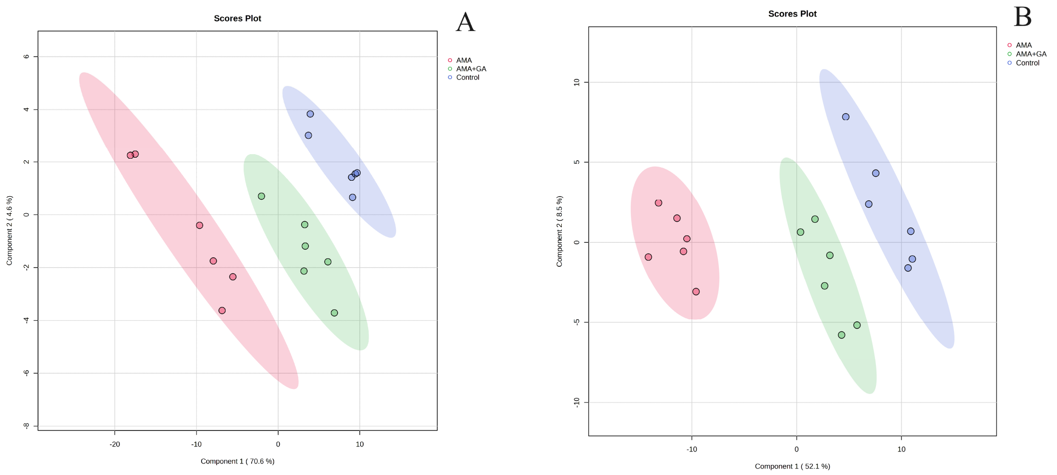

Figure S1. PLS-DA (supervised) analysis of serum metabolites in healthy mice (Control), α-amanitin-exposed mice (AMA) and GA treatment (AMA+GA). (A) Score plot after 24 hours of GA treatment ($R^2 = 0.998$, $Q^2 = 0.935$). (B) Score plot after 48 hours of GA treatment ($R^2 = 0.999$, $Q^2 = 0.903$).

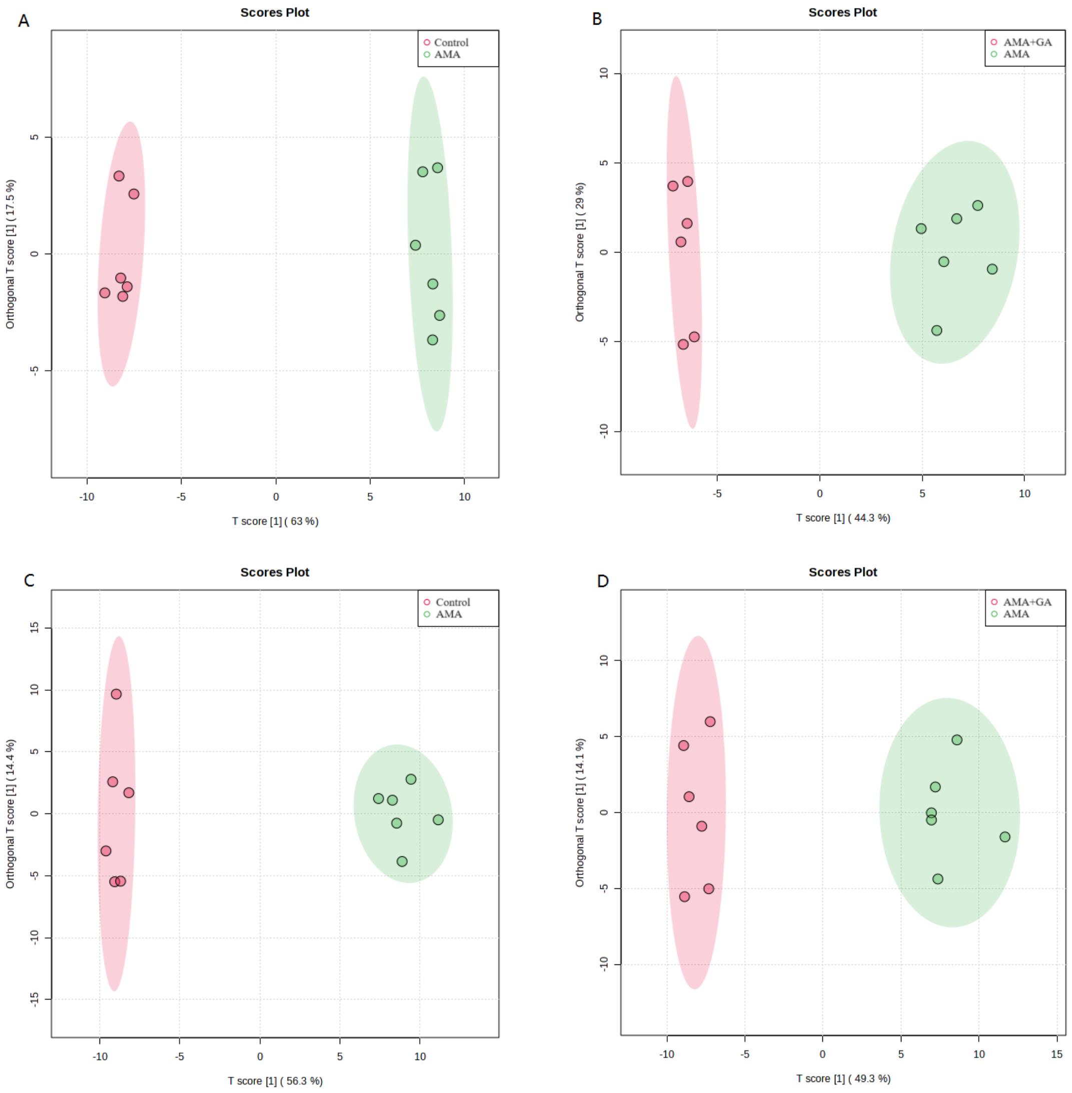

Figure S2. The Orthogonal Partial Least Squares Discriminant Analysis (OPLS-DA) score plots of serum metabolites in healthy mice (Control), α-amanitin-exposed mice (AMA) and GA treatment (AMA+GA). (A) Score of the 24h control versus AMA ($R^2X = 0.639$, $R^2Y = 0.849$ and $Q^2 = 0.815$). (B) Score of the 24h AMA versus AMA+GA ($R^2X = 0.443$, $R^2Y = 0.676$ and $Q^2 = 0.623$). (C) Score of the 48h control versus AMA ($R^2X = 0.563$, $R^2Y = 0.952$ and $Q^2 = 0.930$). (D) Score of the 48h AMA versus AMA+GA ($R^2X = 0.493$, $R^2Y = 0.933$ and $Q^2 = 0.906$).

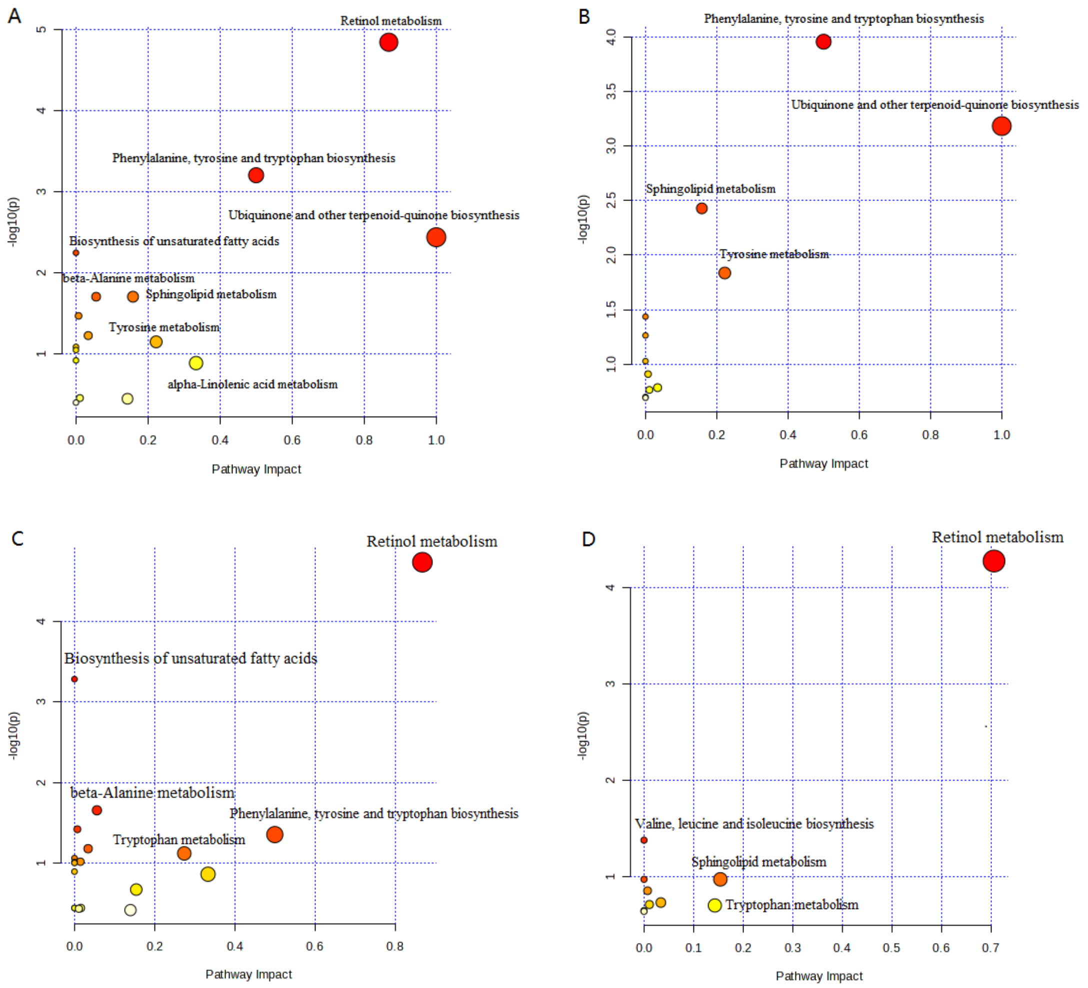

Figure S3. Metabolic pathway analysis results. (A) 24h Control versus AMA. (B)24h AMA versus AMA+GA. (C) 48h Control versus AMA. (D) 48h AMA versus AMA+GA.
